# DNAvisualization.org: a serverless web tool for DNA sequence visualization

**DOI:** 10.1093/nar/gkz404

**Published:** 2019-06-06

**Authors:** Benjamin D Lee, Michael A Timony, Pablo Ruiz

**Affiliations:** 1 In-Q-Tel Lab41, 800 El Camino Real, Suite 300, Menlo Park, CA 94025, USA; 2 Harvard Medical School, 77 Avenue Louis Pasteur, Boston, MA 02115, USA; 3 School of Engineering and Applied Sciences, Harvard University, 29 Oxford Street, Cambridge, MA 02138, USA; 4 SBGrid Consortium, Harvard Medical School, 250 Longwood Avenue, SGM114, Boston, MA 02115, USA

## Abstract

Raw DNA sequences contain an immense amount of meaningful biological information. However, these sequences are hard for humans to intuitively interpret. To solve this problem, a number of methods have been proposed to transform DNA sequences into two-dimensional visualizations. DNAvisualization.org implements several of these methods in a cost effective and performant manner via a novel, entirely serverless architecture. By taking advantage of recent developments in serverless parallel computing and selective data retrieval, the website is able to offer users the ability to visualize up to thirty 4.5 Mb DNA sequences simultaneously using one of five supported methods and to export these visualizations in a variety of publication-ready formats.

## INTRODUCTION

As DNA sequencing technology becomes more commonplace, tools for the analysis of its data are among the most cited papers in science (1). The reason is simple: DNA sequences are, by themselves, almost completely unintelligible to humans. Seeing meaningful patterns in DNA sequences (which are often too large to be shown in their entirety on a screen) is a significant challenge for researchers. Numerous tools, ranging from genome browsers (2) to multiple sequence alignment viewers (3) and dot plot visualizers (4) have been developed to enable interactive browser-based visualization of DNA sequences, alignments, and annotations. A different approach to addressing this problem is to convert DNA sequences directly into two-dimensional visualizations that capture some aspect of the biological information contained within, without relying on external information such as annotations. This approach has the benefit of taking advantage of the highly developed human visual system, which is capable of tremendous feats of pattern recognition and memory (5).

A variety of methods have been proposed to convert DNA sequences into two dimensional visualizations. One common technique is to map each nucleotide to a vector and connect those vectors tip-to-tail to represent a DNA sequence. For example, the Gates method (6) uses up, down, left, and right vectors of length one to represent Ts, As, Cs, and Gs, respectively, while the Yau method (7) uses vectors along a unit circle to represent the bases. Others, such as Qi (8) and its derivative Squiggle (9) algorithm are based on mapping a binary representation of the sequence to upward- and downward-oriented vectors for 1s and 0s, respectively. In contrast, other algorithms such as (10) and (11) are based on tablature, with the x coordinate corresponding to base number and the y coordinate to a specific nucleotide or dinucleotide, respectively. These methods are highly heterogenous, but, for the sake of this paper, we will only discuss methods with no degeneracy, i.e. methods that produce visualizations which may be unambiguously transformed back into the DNA sequences from which they were generated. All of these methods operate on a single underlying principle: they map each nucleotide in a DNA sequence to one or more points in the Cartesian plane and treat each sequence as a walk between these points.

One effect of mapping each base to at least one point is that the number of points grows linearly with the length of the DNA sequence. This poses a technological challenge, as the ability to sequence DNA has vastly outpaced tools to visualize it. Indeed, there is currently a dearth of DNA visualization tools capable of implementing the variety of methods that have been introduced in the literature (9,12,13). Taking inspiration from DNAsonification.org (14), which allows for the auditory inspection of DNA sequences, we propose DNAvisualization.org to fill this gap in the web-based visualization toolset.

## METHODS AND RESULTS

### Interface

The user interface for the tool is deliberately simple. A user first selects one or more visualization methods from the five currently implemented by the Squiggle package (6,7,9,10). The user then provides FASTA-formatted sequence data to visualize, either by using the operating system’s file-input prompt, dragging-and-dropping files onto the browser window, or pasting the raw data into a text prompt. Upon receipt of sequence data, a loading spinner indicates that the system is processing the data. After the data processing is complete, the loading spinner is replaced with the two-dimensional visualization.

**Figure 1.**
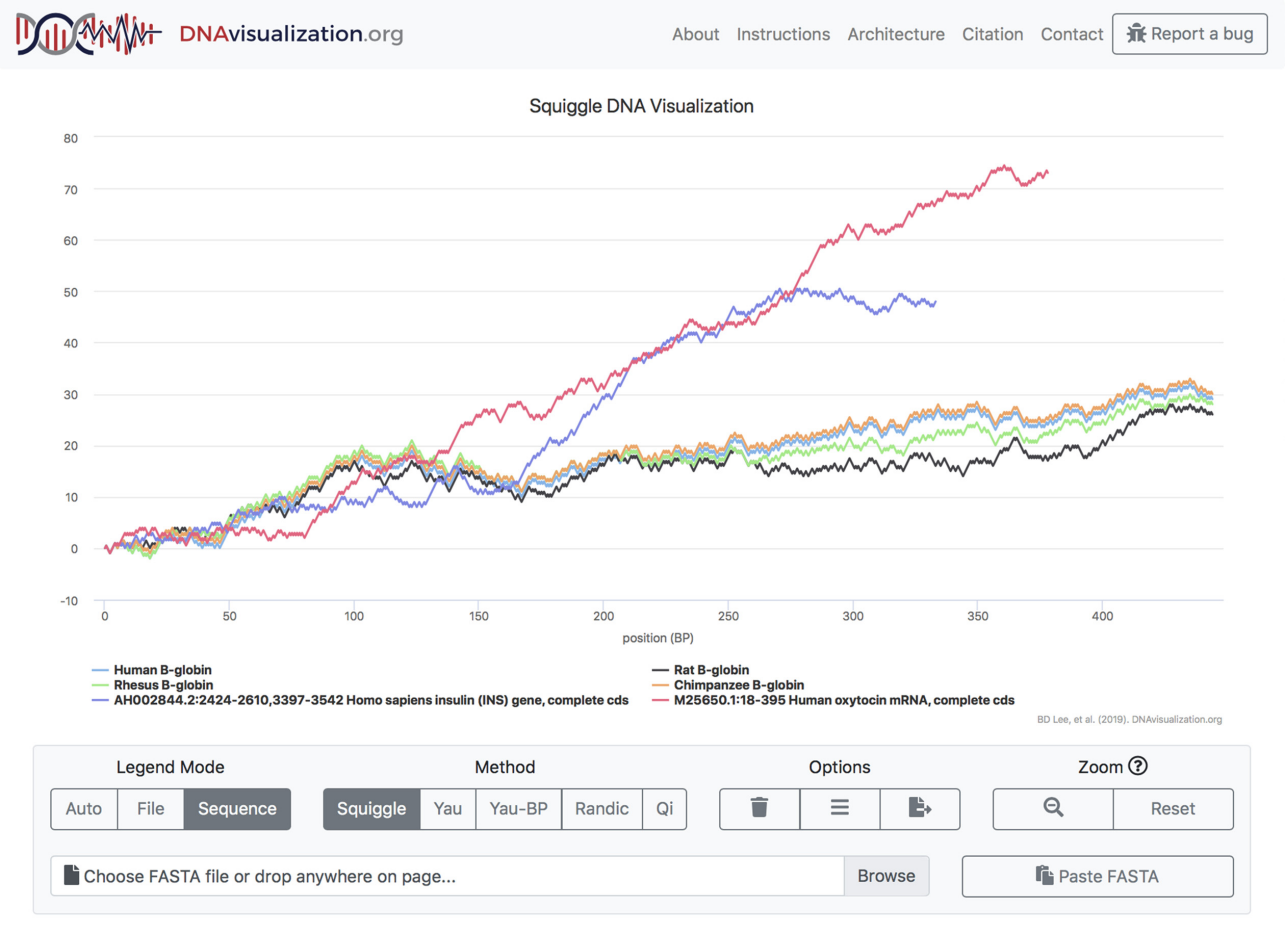
Sequence mode.

**Figure 2.**
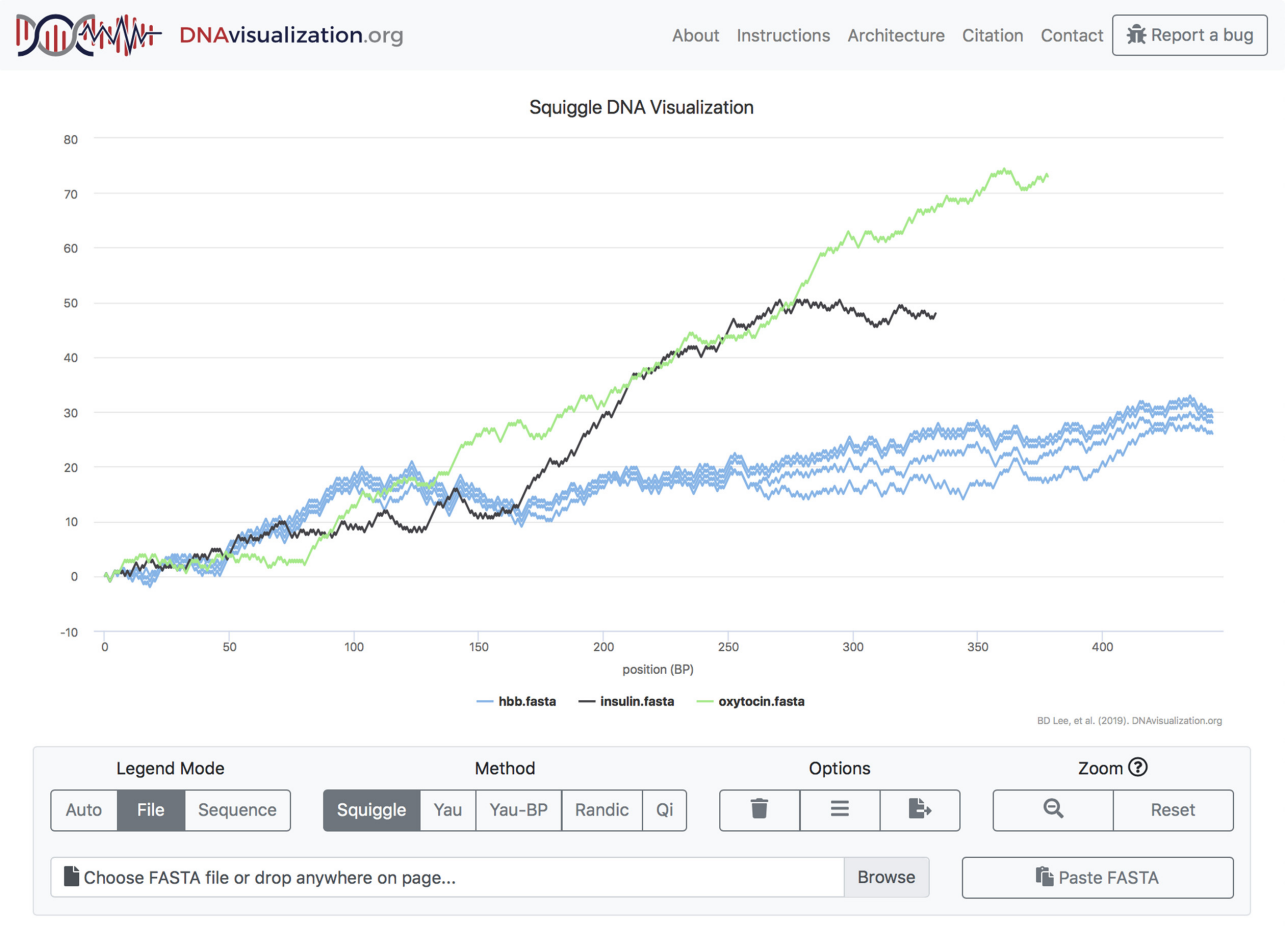
File mode.

The initial view is such that the entirety of each sequence’s visualization is visible: every part of every sequence can be seen. This poses an immediate challenge, as comparing sequences of vastly different lengths will result in the smaller sequence being so small as to be essentially invisible. To solve this problem, the tool allows users to toggle the visibility of sequences by clicking on the corresponding legend entry, which will automatically rescale the visualization’s axes to fit the displayed sequences. The legend coloring is dynamic as well. The user may decide to color code the legend either with each sequence (shown in Figure 1) or each file in its own color (shown in Figure 2) and toggle between options after the data has been plotted, allowing for both inter- and intra-file comparisons.

To inspect a region of the visualization more closely, a user may click and drag over it to zoom in. When zooming in, a more detailed visualization is shown by asynchronously retrieving data for the region, allowing for base-pair resolution analysis. With a single click, the axis scaling may be reset to the default zoom level.

The title and subtitle of the visualization are dynamically set but may be overridden at any time by the user. If the user wishes, their visualization may be downloaded in one of several formats suitable for publication such as SVG, PDF, JPG and PNG.

DNAvisualization.org supports color coding each sequence or file individually.

### Implementation

The web tool is built using a novel architecture, with computing, as well as data storage and selective retrieval, done in an entirely serverless manner. To understand how this system differs from a traditional architecture, consider a traditional approach to building the DNAvisualization.org tool. A server, usually running Linux or Microsoft Windows, is established to handle HTTP requests to the website. This server is either maintained by a university or, increasingly often, a cloud service provider. If there are no requests (as can be expected to be a nontrivial fraction of the time for low-traffic web tools), the server sits idle. When requests are submitted, the server responds to each one. If the server is at capacity, requests may go unanswered or, with additional complexity, more servers may be requested from cloud services provider to meet the greater demand. Data storage is usually provided by a relational database management system (RDBMS), which must also be running on a server.

This paradigm has several disadvantages: disruptions to the server result in disruptions to the website, greater expertise is required for the development and maintenance of the website, the server wastes resources while sitting idle, and the server’s computational and storage capacity is directly limited by its hardware.

A new model has been introduced called serverless computing or Function-as-a-Service (FaaS) that is able to solve these problems. The basic idea is that a software developer specifies code to be executed (i.e. a function) and then invokes it on varying inputs. In fact, the name ‘serverless computing' is a misnomer: the computation still occurs on a server, just not one the developer is responsible for managing. Instead, the cloud service provider is delegated the responsibility for the execution of the code, thus enhancing developer productivity (15). In this model, the pricing is calculated by function invocation, typically metered to the tenth of a second. When not being used, there is no cost to the user. On the other hand, if there are numerous simultaneous function invocations, each invocation is handled separately, in parallel.

By making the serverless function a virtual ‘server' and invoking the function upon each individual request, one is able to take full advantage of serverless computing. For each request to the website, a virtual server is created for just long enough to respond to the request and then immediately extinguished. This results in the website being able to instantly scale to use precisely the resources needed to meet demand.

At the time of this writing, there are a variety of serverless computing platforms including (but certainly not limited to) Amazon Web Services (AWS) Lambda (https://aws.amazon.com/lambda/), Google Cloud Functions (http://cloud.google.com/functions/), and Microsoft Azure Functions (https://azure.microsoft.com/en-us/services/functions/), each of which differ in terms of factors such as supported programming languages, startup latency, and pricing structure (16). DNAvisualization.org is built atop AWS Lambda due to its permanent free tier that, at the time of this writing, allows for one-million free function invocations totaling up to 3.2 million seconds of compute time per month, which is anticipated to easily meet the demand for the site. In the event that the free tier is exceeded, AWS Lambda’s pricing is $0.20 per million function invocations and $0.00001667 dollars per GB-second of computation (one GB-second corresponds to using a Lambda function with 1 GB of RAM for one second) at the time of this writing.

For DNAvisualization.org, we use AWS Lambda to serverlessly transform submitted DNA sequences into their visualizations in parallel, in addition to serving the static assets (*i.e*. HTML, Javascript, and CSS files) to the user. The site uses Python’s Flask web microframework (http://flask.pocoo.org) and has its deployment to AWS Lambda seamlessly automated by the Zappa tool (https://github.com/Miserlou/Zappa).

It must be noted that using a serverless architecture to host a website is not novel by itself. Rather, the novelty of the architecture lies in its combination of serverless computing for request handling with query-in-place data retrieval on compressed data. As mentioned previously, a normal web architecture would use a server running a RDBMS to handle data storage. In the case of DNA visualization, the database would be used to persist the transformed DNA sequences as x- and y-coordinates that may be queried when zooming in on a region. However, using a database server creates many of the same issues as using a server for web hosting, such as scalability, cost, and parallelism. Instead of using an RDBMS, we used the S3 cloud storage platform combined with the S3 Select query-in-place functionality offered by AWS. In essence, this service allows one to upload a compressed tabular file to S3 and then submit a SQL query to be executed against the tabular data. In this paradigm, pricing is based on the amount and duration of data storage, the amount of data scanned during querying, and the amount of data returned by query. At the time of this writing, the price of storage in S3 is $0.023 per GB per month (for the first 50 TB of data), with S3 Select priced at $0.002 per GB of data scanned and $0.0007 per GB of data returned.

For DNAvisualization.org, each submitted sequence’s transformation is stored on AWS S3 in the open-source Apache Parquet tabular data format using Snappy columnar compression. Then, when a user zooms in on a region, a request is sent to AWS Lambda, which submits a SQL query to S3 Select, which in turn scans the file for data in the region. The matching data are then returned to the Lambda function, which downsamples the data if necessary (to prevent wasting users’ memory with more data points than can be shown) and returns it to the browser, which then updates the visualization. This process happens entirely in parallel for each sequence that the user has submitted, regardless of how much demand there is on the website, showcasing the usefulness of serverless computing. The S3 buckets (i.e. folders) containing the cached DNA sequence transformations are configured such that twenty-four hours after a user has submitted a sequence for visualization, its transformation is automatically deleted, thereby further reducing the cost of the website’s operation.

**Figure 3.**
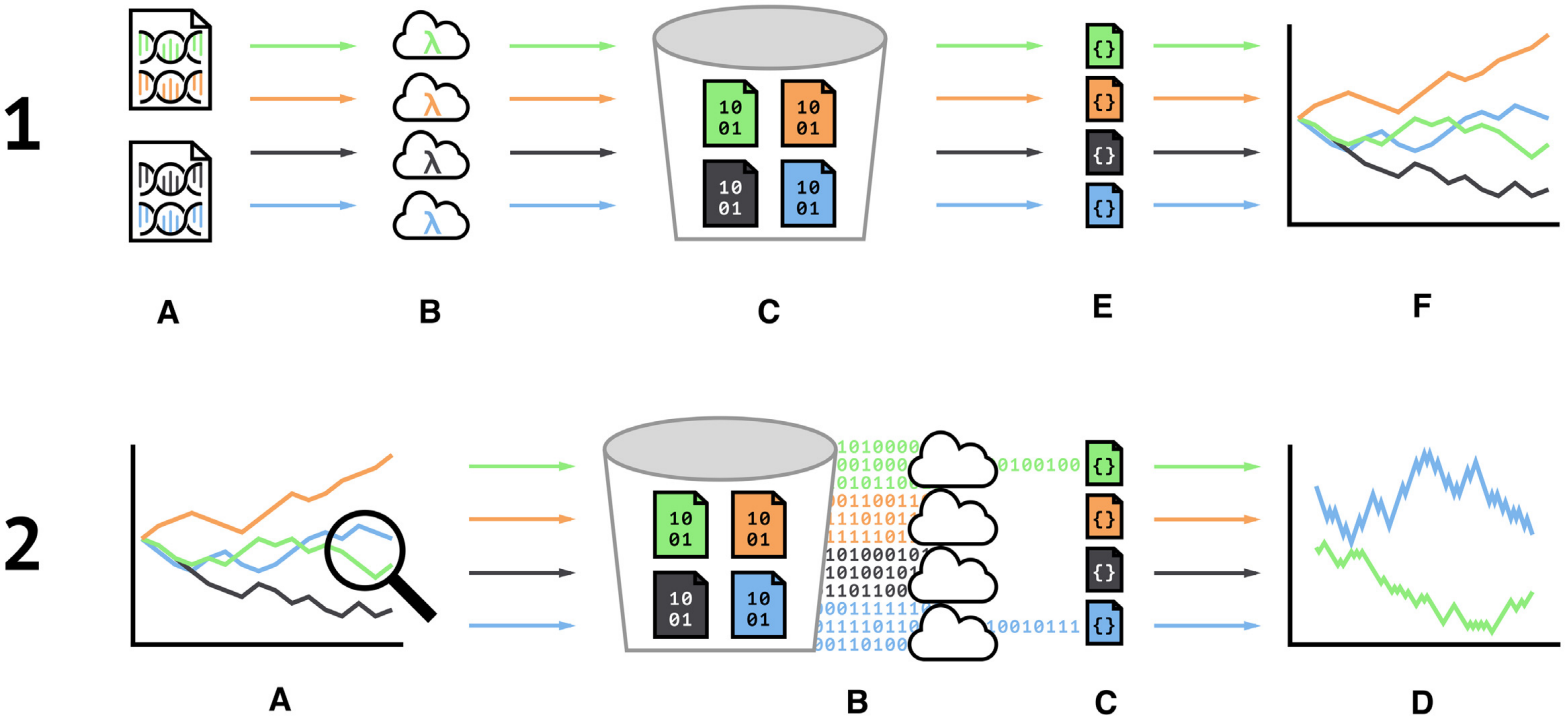
A diagram displaying the flow of information during initial sequence transformation (1) and sequence querying (2). For initial sequence transformation, FASTA files (1A) are parsed in the user’s browser and submitted asynchronously in parallel to the serverless Lambda functions (1B). If the sequence does not already have an existing transformation using the specified visualization method, the functions transform the sequence and store the data in AWS S3 (1C) in the binary Parquet format. The functions then downsample the transformed data and return it the client’s browser in JSON format (1E) to be plotted (1F). If a user wishes to see more detail of a particular region (2A), the browser sends an asynchronous query containing the location of the region for each plotted DNA sequence to a Lambda function (not shown). In parallel, each function converts the query into a SQL statement and submit the query to S3 Select (2B). S3 Select scans the transformed DNA sequence and returns only the data in the region to the Lambda function, which in turn downsamples to JSON (2C) and returns it to the user’s browser for plotting (2D).

An overview of the architecture is presented in Figure 3.

## DISCUSSION

Because DNA sequence transformation is an inherently parallelizable task, the use of serverless computing is a natural fit for this application. However, not all web applications for biology are currently amenable to serverless computing due to the constraints imposed by cloud services providers.

The primary limitation of serverless computing for web tools is the necessity for a short duration of computation (currently on the scale of seconds to minutes, depending on the platform) or, failing that, the ability to parallelize the computation and the data. In addition, a function’s memory use may not exceed a predefined limit, which can range from the scale of megabytes to several gigabytes and be specified by the user. Applications which violate these requirements will need significant modifications to this architecture in order to function. An example of a web server that cannot trivially be ported to use an entirely serverless architecture is MISTIC2 (17), a tool for protein coevolution analysis, whose job durations can be as long as five hours. This job length is significantly longer than any current serverless offering allows a single function invocation to run. However, as the capabilities of serverless computing increase, the burden of these limitations will decrease. For more information about the limitations of serverless computing, see (15) and (18).

These limitations were bypassed by this tool in several ways, which may be of interest to readers attempting to implement similar architectures in the future. When implementing parallelization, we were faced with a choice between higher, file-level parallelization (parsing and transforming each file’s sequences in a separate Lambda function invocation) and lower, sequence-level parallelization (parsing the files in the browser and invoking a Lambda function to transform each sequence individually). We initially chose the former but quickly ran into memory issues, even when opting to use the most generous memory allocation available (3008 MB at the time of writing, which includes all of the function’s code as well as the data on which it is invoked). To reduce memory demands, we switched to sequence-level parallelism and eliminated as many dependencies as possible. While this tradeoff results in increased memory use by the client, which must load and parse the FASTA files in memory, and greater cost because pricing is by both the function invocation and the total amount of computation (which remains the same, as the total number of bases that must be transformed does not change), it enables greater throughput by more effectively leveraging parallelism.

Currently, the website is limited to visualizing up to thirty sequences of up to 4.5 Mb each for a grand total of 135 Mb of sequence data at a time. The total sequence count limitation ensures that our chart renderer can handle rendering all of the points (downsampled to a static 1000 points per sequence) and the sequence length limitation ensures that the transforming Lambda function’s memory is not overwhelmed. In the future, we aim to increase this limit by taking advantage of further optimizations in memory management during transformation and increases in the total amount of memory available to function invocations.

While this website was implemented using AWS, this architecture is not exclusive to AWS. Google Cloud Platform (GCP) offers both serverless computing and serverless data querying via their BigQuery platform. Although both GCP and AWS have similar offerings, it is nontrivial to change cloud service providers due to each service provider’s use of a proprietary application programming interface (API). The issue of vendor lock-in via proprietary APIs is one of the open problems in serverless computing (15), although open-source tools such as the Serverless Framework (https://www.github.com/serverless/serverless) and Apache OpenWhisk (https://openwhisk.apache.org) show promise for ameliorating this issue.

## CONCLUSION

This web tool serves as a demonstration of the applicability of serverless computing to computational molecular biology as well as a useful tool to quickly gain an intuitive visual overview of DNA sequences. While not all applications are amenable to serverless computing, those that are may achieve greater performance with decreased cost and development complexity, a significant advantage over traditional web architectures. By making sequence visualization fast and simple as well as by providing an open-source example of serverless computing and data retrieval, this tool aims to make both of these valuable techniques more widely used within the biological research community.

## DATA AVAILABILITY

The website is freely accessible at https://DNAvisualization.org. The software repository is hosted at https://github.com/Benjamin-Lee/DNAvisualization.org.
